# Adipocytes Under Obese-Like Conditions Change Cell Cycle Distribution and Phosphorylation Profiles of Breast Cancer Cells: The Adipokine Receptor CAP1 Matters

**DOI:** 10.3389/fonc.2021.628653

**Published:** 2021-03-02

**Authors:** Malin Bergqvist, Karin Elebro, Signe Borgquist, Ann H. Rosendahl

**Affiliations:** ^1^ Department of Clinical Sciences Lund, Oncology, Lund University, Skåne University Hospital, Lund, Sweden; ^2^ Department of Clinical Sciences Malmö, Surgery, Lund University, Skåne University Hospital, Malmö, Sweden; ^3^ Department of Clinical Medicine, Aarhus University, Aarhus, Denmark; ^4^ Department of Oncology, Aarhus University Hospital, Aarhus, Denmark

**Keywords:** adipocyte, obesity, breast cancer, CAP1, protein phosphorylation, proliferation, cell cycle

## Abstract

**Background:**

Obesity and associated metabolic conditions impact adipocyte functionality with potential consequences for breast cancer risk and prognosis, but contributing mechanisms remain to be understood. The adipokine receptor adenylyl cyclase-associated protein-1 (CAP1) has been implicated in the progression of breast cancer, but results are conflicting and the underlying molecular mechanisms are still unknown. In this study, molecular and cellular effects in breast cancer cells by stimulation of adipocytes under normal or obese-like conditions, and potential involvement of CAP1, were assessed.

**Material and Methods:**

Estrogen receptor (ER)-positive T47D and ER-negative MDA-MB-231 breast cancer cells were exposed to adipocyte-secretome from adipocytes placed under pressures mimicking normal and obese-like metabolic conditions. Changes in phosphorylated kinase proteins and related biological pathways were assessed by phospho-antibody array and PANTHER analysis, cell proliferation were investigated through sulforhodamine B, cell cycle distribution by flow cytometry. Functional effects of CAP1 were subsequently examined following small interfering (si)RNA-mediated knockdown.

**Results:**

Protein phosphorylations involved in important biological processes were enriched in T47D breast cancer cells in response to adipocyte secretome from obese-like compared with normal conditions. The obesity-associated adipocyte secretome further stimulated cell proliferation and a shift from cell cycle G1-phase to S- and G2/M-phase was observed. Silencing of CAP1 decreased cell proliferation in both T47D and MDA-MB-231 cells, and reduced the obesity-associated secretome-induction of phosphoproteins involved in cell proliferation pathways.

**Conclusions:**

These results indicate that the adipocyte secretome and CAP1 are mechanistically important for the proliferation of both ER-positive and ER-negative breast cancer cells, and potential signaling mediators were identified. These studies provide biological insight into how obesity-associated factors could affect breast cancer.

## Introduction

Obesity is increasingly common and its worldwide prevalence has nearly tripled since 1975 (1). With almost two billion adults being overweight (body mass index; BMI ≥25 kg/m^2^), and 650 million hereof being obese (BMI ≥30), in 2016 (1), excess body fat is recognized as one of the most important public health issues as of today. Obese individuals are further at increased risk of developing other diseases, including breast cancer (2), where elevated body fatness and altered metabolism have been shown to favor both breast tumor initiation and progression (3–5).

The combination of energy-rich food and sedentary lifestyle leads to an expansion of adipose tissue as adipocytes increase in size (hypertrophy) and number (hyperplasia), resulting in overweight or obesity (6). Adipocyte hypertrophy can result from accumulation of triglycerides and have a suggested role in the development of insulin resistance and altered glucose homeostasis (7). Hyperglycemia can occur in both obesity and type 2 diabetes due to gluconeogenesis and an impaired ability of insulin to inhibit glucose output from the liver, and to promote glucose transport (8). Insulin resistance, a major feature of both the metabolic syndrome and type 2 diabetes has been associated with increased mortality for women with breast cancer, possibly related to the growth-promoting effects of insulin, insulin-like growth factors (IGF), and to hyperglycemia (9, 10). Hyperinsulinemia caused by insulin resistance can activate mitogenic pathways and inhibit cell apoptosis, contributing to a carcinogenic environment (5). Several studies have shown that breast cancer patients with hyperglycemia, insulin resistance, and hyperinsulinemia have a higher relapse rate and mortality compared to breast cancer patients with normal metabolic conditions (11–13).

Breast cancer is the most common cancer among women and the leading cause of cancer death in females worldwide (14). In postmenopausal women, obesity is an established primary risk factor for estrogen receptor (ER)-positive breast cancer (15). The increased risk is, at least in part, proposed to be attributed to increased levels of estrogen in obese women due to excessive adipose tissue (16). At menopause, the ovaries cease to produce hormones and the primary source of estrogen is based on aromatase activity in adipose tissue. For obese postmenopausal woman, elevated aromatase activity and the resulting higher estrogen exposure may fuel the growth of estrogen-responsive breast tumor (16). Obesity may further increase the risk of ER-negative or triple-negative breast cancer in pre-menopausal women (17). In addition to the increased breast cancer risk, obese breast cancer patients also have a worse prognosis and outcome irrespective of menopausal or hormone receptor status, compared with breast cancer patients of normal weight (18).

Obesity affects breast cancer development and progression in several ways. Apart from synthesizing estrogen, the adipose tissue functions as an endocrine organ, secreting adipokines and inflammatory cytokines (5, 19). The inflammatory adipokine resistin is elevated in obesity and has an important role in insulin resistance (20–22). High circulating resistin levels have been associated with cancer stage, histological grade, tumor size, lymph node involvement, and poor clinical outcome in breast cancer (23–26). A preclinical study demonstrated that adipocytes under obesity-associated metabolic conditions have an increased secretion of resistin (27). The highly conserved actin-binding protein adenylyl cyclase-associated protein 1 (CAP1) serve as a receptor for resistin (28). CAP1 is an ubiquitously expressed protein involved in cytoskeletal rearrangements in most tissues (29, 30). The cytoskeleton plays a vital role in cancer development and progression as it contributes to the regulation of both cell division and motility (31). In experimental models, CAP1 knockdown has been demonstrated to disrupt the actin cytoskeleton, supporting a role for CAP1 in actin turnover (29, 30, 32). While some studies show a decreased cell proliferation and migration, and poor prognosis for depleted/low CAP1 tumors, others report the opposite (27, 32, 33). Thus, the role of CAP1 in breast cancer is still controversial.

Further molecular mechanistic insights are needed to help improve the understanding of how breast cancer cells respond to an altered host energy balance and a modified adipocyte functionality under obese-like conditions. We hypothesized that the adipocyte secretome, placed under pressure mimicking obese metabolic conditions, would promote cell cycle progression and induce signaling pathways involved in proliferation and motility, and that CAP1 silencing would reduce these obesity-induced changes. The aim of this study was to investigate the impact of CAP1 and obesity on breast cancer cell proliferation, cell cycle distribution, and protein phosphorylation patterns to gain a better understanding of how adipokines affect breast cancer cells.

## Material and Methods

### Reagents

All chemicals and reagents were purchased from Sigma Aldrich (Saint Louis, Missouri, USA) unless stated otherwise. Cell culture media, penicillin/streptomycin, insulin, NuPAGE gels, and MOPS buffer were purchased from Invitrogen (Carlsbad, California, USA). Bovine calf serum (BCS) was purchased from ATCC-LGC Standards (Manassas, Virginia, USA). Fetal bovine serum (FBS) and PBS were from HyClone (GE Healthcare, Chicago, Illinois, USA). Scrambled or anti-CAP1 small interfering RNA (siRNA), Pierce BCA Protein Assay Kit, and SuperSignal West Dura Extended Duration Substrate were purchased from ThermoFisher Scientific (Waltham, Massachusetts, USA). NP-40 Alternative was purchased from VWR (Radnor, Pennsylvania, USA). Proteome Profiler Human Phospho-Kinase Array Kit (ARY001B) was purchased from R&D Systems (Minneapolis, Minnesota, USA).

### Cell Culture

Human breast cancer cell lines, ER-positive T47D and ER-negative MDA-MB-231, and pre-adipocyte fibroblast cell line, 3T3-L1, were purchased from ATCC. The breast cancer cells were maintained in Dulbecco’s modified Eagle’s Medium (DMEM) supplemented with antibiotics (100 U/ml penicillin and 100 µg/ml streptomycin), and 10% FBS (breast cancer cells) or 10% BCS (3T3-L1 cells), and grown at 37°C in humidified 5% CO_2_ atmosphere. Pre-adipocytes grown to confluency were differentiated in DMEM with antibiotics, 10% FBS, 1 µM dexamethasone, 0.5 mM methylisobutylxanthine (IBMX), and 1 µg/mL insulin for 48h. The differentiation medium was subsequently replaced with maintenance medium DMEM supplemented with antibiotics, 10% FBS, and 1 µg/mL insulin. Full adipocyte differentiation was obtained within 7–14 days.

### Normal- and Obesity-Associated Adipocyte Conditioned Medium

**Figure 1:**
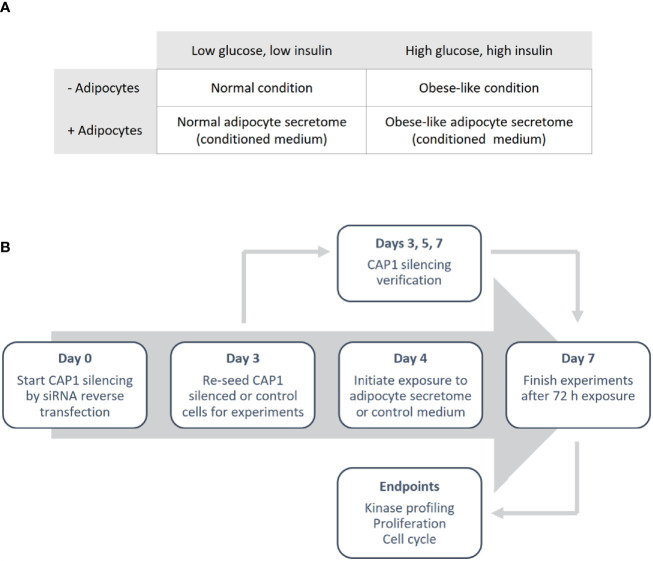
Outline of the study design. **(A)** Overview of the different metabolic conditions, in the absence and presence of adipocytes, used in the study. **(B)** Schematic illustrating the experimental process.

Differentiated adipocyte were cultured for 24 h in normal condition (low glucose; 5 nM and low insulin; 0.1 ng/mL) or obese-like condition (high glucose; 25 nM and high insulin; 1 µg/mL) in a serum-free medium (SFM: DMEM supplemented with transferrin; 0.04 mg/mL, sodium bicarbonate; 1.2 mg/mL, BSA 0.2 mg/mL and antibiotics) to generate adipocyte secretome-containing conditioned medium, and in parallel control medium (adipocyte-absent) were collected (**Figure 1A**). The medium was centrifuged to remove cell debris and used immediately.

### CAP1 siRNA Knockdown

Breast cancer cells were reverse transfected with anti-CAP1 or scrambled (non-targeting control) siRNA. In brief, cells were seeded in a 6-well plate to obtain 70% confluence in a transfection solution consisting of 1.25 mL Opti-MEM and 1.25 mL full culture medium (antibiotics excluded), 10 µL Lipofectamine 2000 and 25 nM CAP1 siRNA (T47D: Silencer^®^ Select s20547 and Silencer^®^ Select s20549; MDA-MB-231: Silencer^®^ Select s20547) or Silencer^®^ Select negative control No 1. Following 72 h incubation (day 3), the CAP1 silenced or control cells were washed, harvested and re-seeded for further experiments. An outline of the study design is presented in **Figure 1B**.

### Western Immunoblotting

Breast cancer cell lysates were prepared from control and *CAP1* knockdown cells cultivated for 3, 5, and 7 days. To prevent confluency, the cells were sub-cultured at day 3 post siRNA transfection. Proteins were extracted using radioimmunoprecipitation assay buffer (RIPA; 10 mM Tris–HCl pH 7.4, 50 mM NaCl, 5 mM EDTA, 30 mM sodium pyrophosphate, 50 mM sodium fluoride, 100 µM sodium orthovanadate, 1% Triton X-100) supplemented with protease and phosphatase inhibitors. Protein concentration was determined using Pierce BCA Protein Assay Kit according to the manufacturer’s instructions. Protein lysates were dissolved in 4x Laemmeli buffer, boiled for 5 min at 95°C and separated (10–20 µg per lane) by SDS-PAGE (NuPage 10 or 8–12%) and transferred to nitrocellulose membranes by Trans-Blot Turbo Transfer System (Bio-Rad Laboratories, Hercules, California, USA). The membranes were blocked with 5% (w/v) non-fat dry milk in Tris buffered saline with Tween-20, and probed overnight at 4°C with primary antibodies to CAP1 (Abcam; ab133655, 1:10,000) or GAPDH (Sigma Aldrich; MAB374, 1:1,000). Proteins were visualized using HRP-conjugated secondary antibodies and SuperSignal West Dura Extended Duration Substrate, using FluorChem^®^ FC2 imaging system (Alpha Innotech, San Leandro, California, USA) and AlphaView version 3.0.3.0 software (ProteinSimple, Minneapolis, Minnesota, USA) or Odyssey Imaging System (LI-COR Biosciences, Lincoln, Nebraska, USA) and Image J software (NIH, Bethesda, Maryland, USA).

### Cell Proliferation

Breast cancer cells, scrambled and anti-CAP1 siRNA treated, were seeded in 96-well plates and grown in complete growth medium. After 24 h the cells were washed in PBS, starved in SFM for 6 h, and then exposed to control or adipocyte secretome-containing conditioned medium from normal or obese-like metabolic conditions for 72 h. Cell growth was measured by Sulforhodamine B (SRB) assay. Briefly, cells were fixed in ice-cold 17% trichloroacetic acid and stained with 0.4% SRB in 1% acetic acid. Unbound SRB was washed away with 1% acetic acid and the protein-bound dye dissolved in 10 nM TRIS base buffer. The absorbance was then quantified on a VersaMax microplate reader at 570 nm using the SoftMax Pro software (Molecular Devices, San Jose, California, USA).

### Cell Cycle Analysis

Breast cancer cells were exposed to adipocyte secretome or control as described above. After 72 h incubation, cells were harvested by EDTA/trypsinization, fixed in ice-cold 70% ethanol, and stored at −20°C until use. Upon analysis, the cells were washed to remove ethanol fixation and stained with Propidium Iodide – Nuclear Isolation Medium (PI-NIM)-RNase (0.05 mg/mL PI, 0.6% (v/v) NP-40 Alternative, 0.1 mg/mL RNase in PBS) for 30 min. The suspension was filtered through a 50 µm nylon filter and DNA content analyzed by flow cytometry (BD Accuri C6, BD Biosciences, Mississauga, ON, Canada). The proportional distribution and discrimination of cells between sub G1, G1, S, G2/M phase was quantified using FlowJo v10.6.1 Software (Franklin Lakes, New Jersey, USA), applying the Watson Pragmatic algorithm for T47D and the Dean-Jett-Fox model with G2-peak (1.89) constraint for MDA-MB-231 for best cell cycle phase fit.

### Proteome Profiler Array

Cellular extracts (200 µg) from CAP1 expressing or CAP1 silenced T47D cells exposed to adipocyte secretome from normal or obese-like conditions for 72 h as described above were incubated onto Proteome Profiler Human Phospho-Kinase Array according to the manufacturer’s instructions. The bound proteins were visualized with Odyssey Imaging System (LI-COR Biosciences, Lincoln, Nebraska, USA). Relative levels of phosphoproteins were normalized against internal references after background subtraction using Image J software (NIH, Bethesda, Maryland, USA). The average density of duplicate spots for each phosphorylated protein kinase was determined, and the fold change in intensity between obese-like relative to normal conditions calculated.

### Biological Process Classification

The Gene Ontology (GO) enrichment analysis tool from the PANTHER Classification System (version 14.0) (34) was used to identify biological processes linked to detected phosphorylation patterns obtained from the Human Phospho-Kinase Array. Enriched GO terms (Biological Process) with a ≥1.25-fold expression change for T47D cells exposed to adipocyte secretome from obese-like relative to normal conditions were reported. Overrepresentation was tested in the PANTHER GO-slim Biological Process data set with the complete Homo sapiens gene list as reference. Fisher’s exact test with calculated false discovery rate (FDR) correction was applied and biological processes with a *P*<0.05 were displayed. Protein interaction network analysis within enriched biological processes were retrieved by the use of STRING version 11.0 (https://string-db.org) (35).

### Statistical Analysis

Western immunoblot, cell proliferation and cell cycle data are presented as mean ± SEM of a minimum of three individual experiments. Multiple comparisons between groups were performed with two-way analysis of variance (ANOVA) with Sidak’s post-hoc test using GraphPad Prism software v8 (San Diego, California, USA). *P*-value of less than 0.05 was considered statistically significant.

## Results

### Obesity-Associated Adipocytes Secretome Affects the Phosphorylation Pattern of Proteins Involved in Cell Proliferation

**Figure 2:**
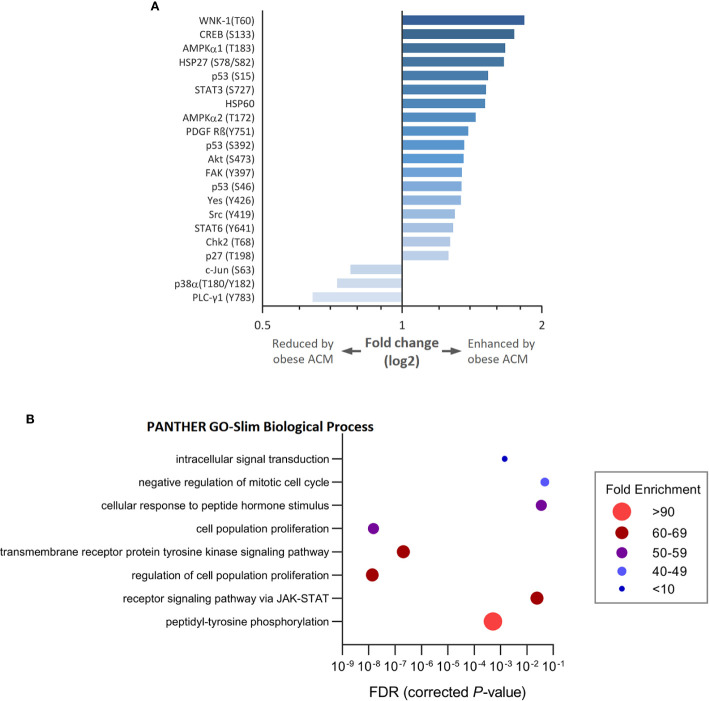
Modulation of kinase phosphorylation profiles and associated biological processes in estrogen receptor positive T47D breast cancer cells in response to adipocyte secretome from obese- compared with normal conditions. **(A)** Graph shows fold changes of phosphorylation pattern in T47D cells following exposure to adipocyte conditioned media (ACM) from obese relative to normal conditions. Phosphorylation patterns modulated ≥ 1.25-fold are shown. **(B)** Bubble plot shows the fold enrichment of biological processes over-represented among enhanced phosphorylated kinases. False discovery rate (FDR) < 5%.

To gain insight into how breast cancer cells recognize and respond to adipocyte secretome from normal or obese-like conditions the phosphorylation profiles of a panel of kinases and associated cell signaling processes were assessed. In total, 43 kinase phosphorylation sites and two related proteins were explored. In ER-positive T47D breast cancer cells, the relative extent of phosphorylation of 18 proteins increased, and three decreased ≥1.25-fold, as a response to secretome stimulation from adipocytes placed under obese-like conditions compared to normal metabolic conditions (**Figure 2A**). Obesity-associated adipocyte secretome increased the phosphorylation of WNK-1 (T60), CREB (S133), AMPKα1 (T183), and HSP27 (S78/S82) more than 1.5-fold compared with normal condition. A relative decrease in c-Jun (S63), p38α (T180/Y182), and PLC-γ1 (Y793) was observed for cells exposed to adipocyte secretome from obese-like compared with normal conditions.

Upregulated phosphoproteins were classified into biological processes according to their GO terms (**Figure 2B**). Enriched processes were most strongly associated with transmembrane receptor protein tyrosine kinase signaling, cell population proliferation, and the regulation thereof. Other altered processes included peptidyl-tyrosine phosphorylation, receptor signaling *via* JAK-STAT, response to peptide hormone stimulus, negative regulation of mitotic cell cycle, and intracellular signal transduction.

### CAP1 Knockdown Duration

**Figure 3:**
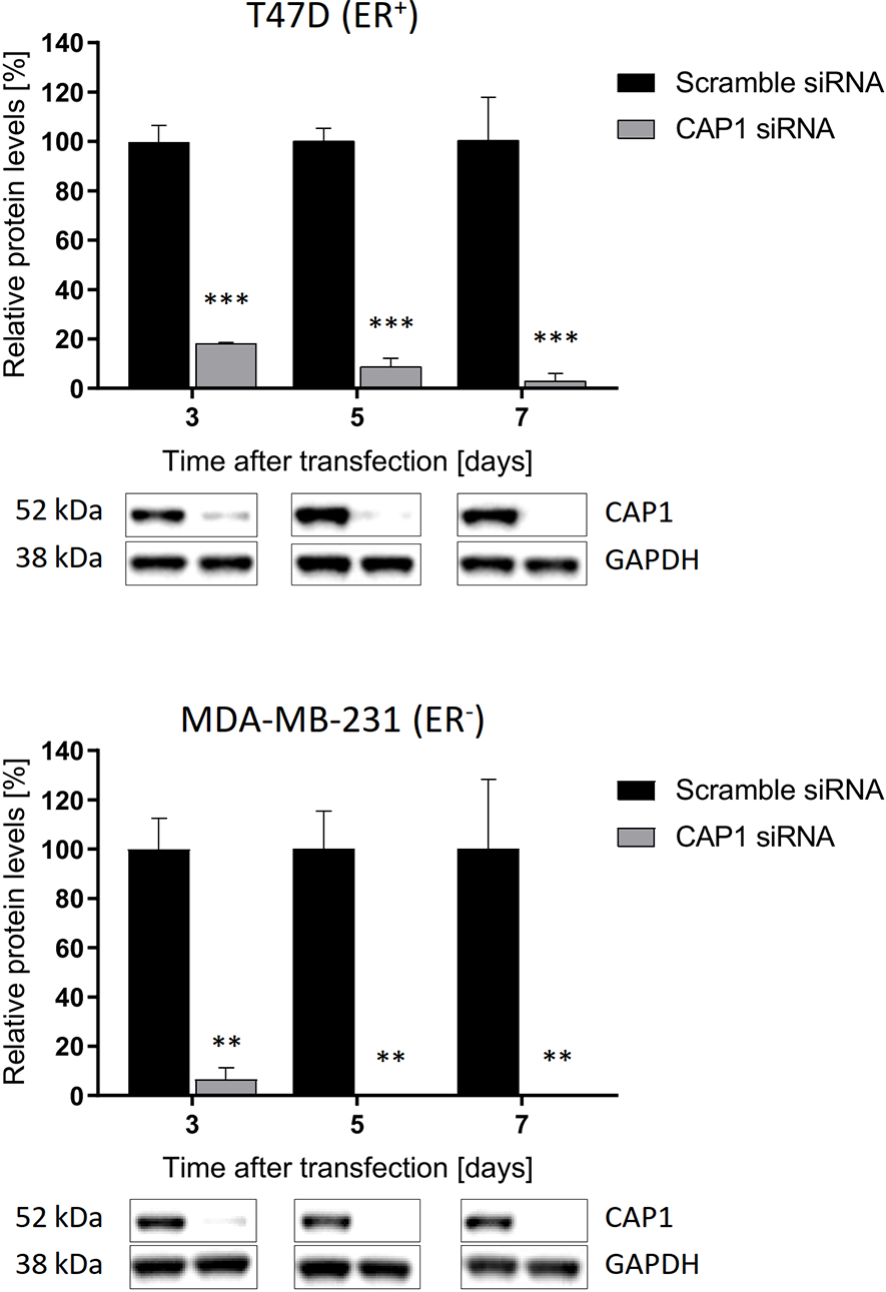
Sustained suppression of CAP1 protein levels following siRNA-mediated knockdown. Images and graphs show CAP1 levels 3, 5, and 7 days after siRNA transfection in ER-positive T47D cells and ER-negative MDA-MB-231 cells. Densitometry analysis of Western immunoblot data was performed and normalized against GAPDH housekeeping protein. Graphs show mean ± SEM from three independent experiments with statistical comparisons between groups by two-way ANOVA with Sidak’s post-hoc test. **P < 0.01; ***P < 0.001.

To assess potential influence by the adipokine receptor CAP1 on the responsiveness to adipocyte-stimulated processes, CAP1 expression was silenced through siRNA-mediated knockdown. Assessment of CAP1 knockdown in the breast cancer cells was evaluated over 3, 5, and 7 days post transfection (**Figure 3**), the time period required for the subsequent experiments. Efficient and sustainable *CAP1* knockdown was obtained over the one-week time course. At day 3 after CAP1 siRNA transfection, T47D and MDA-MB-231 cells displayed 82% (*P*<0.001) and 94% (*P*<0.01) CAP1 protein reduction, respectively. After 7 days, 97% and virtually complete CAP1 protein depletion was verified in T47D (*P*<0.001) and MDA-MB-231 cells (*P*<0.01), respectively.

### CAP1 Knockdown Decreases Adipocyte-Mediated Cell Proliferation

**Figure 4:**
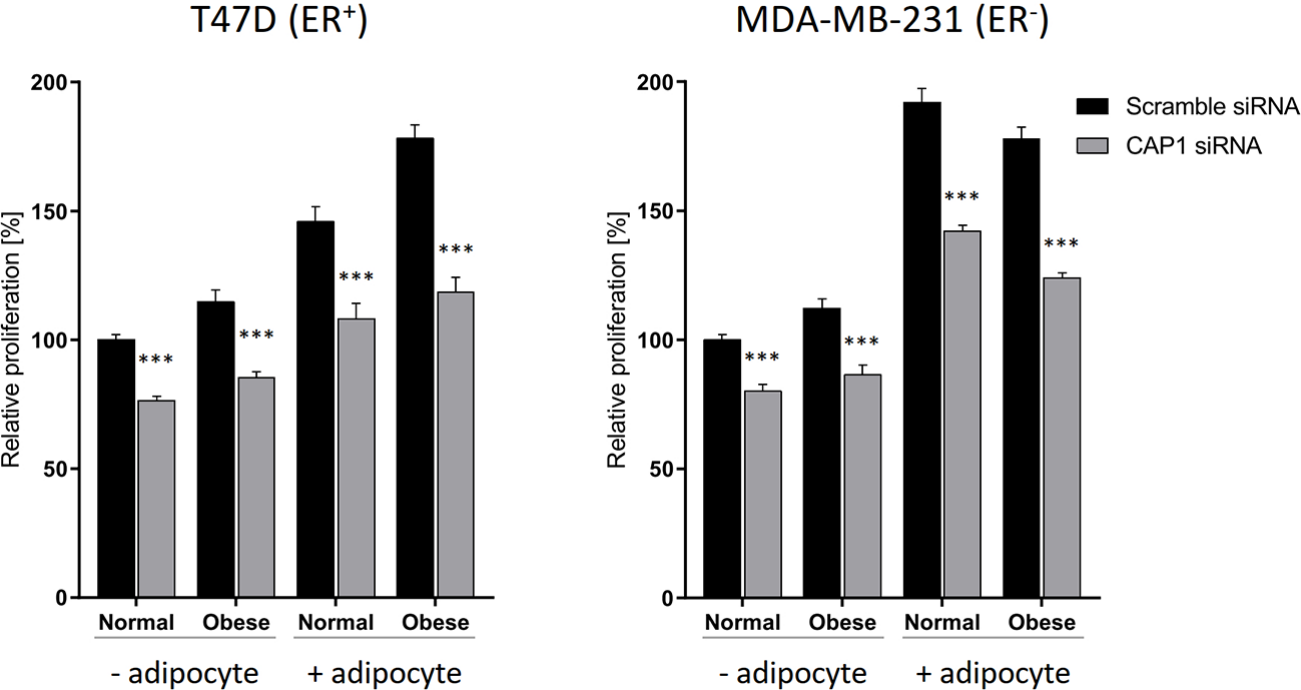
Effect of CAP1 silencing on breast cancer cell proliferation in the presence or absence of adipocyte secretome stimulation under normal and obese-like metabolic conditions in ER-positive T47D cells and ER-negative MDA-MB-231 cells. Three days after CAP1 knockdown, CAP1 silenced or control cells were re-seeded for proliferation experiments, after 24 h the medium was changed to adipocyte secretome or control medium, and proliferation assessed following 72 h exposure. Graphs show mean ± SEM from four to five independent experiments with statistical comparisons between groups by two-way ANOVA and Sidak’s post-hoc test. ***P < 0.001.

The responsiveness to adipocyte secretome with regards to cell proliferation, and to what degree these events involve CAP1 were further assessed. The breast cancer cell proliferation was enhanced in response to adipocyte secretome stimulation, and knockdown of CAP1 in both ER-positive T47D and ER-negative MDA-MB-231 cells decreased proliferation, compared with CAP1 expressing cells (**Figure 4**). In the absence of adipocyte stimulation, CAP1 knockdown resulted in approximately 25 and 20% reduced proliferation of T47D and MDA-MB-231 breast cancer cells, respectively, in both normal and obese-like conditions (*P*<0.001). This decrease was greater in the presence of adipocyte stimulation, compared with control. The adipocyte-induced proliferation under normal conditions was reduced by 26% for both T47D and MDA-MB-231 cells (*P*<0.001) after CAP1 knockdown. In the presence of adipocyte stimulation from obese-like conditions, CAP1 silencing resulted in 33 and 30% reduced proliferation of T47D and MDA-MB-231 cells, respectively (*P*<0.001). No distinct morphological alterations were observed following CAP1 silencing.

### Adipocyte-Derived Factors Alter the Cell Cycle Distribution

**Figure 5:**
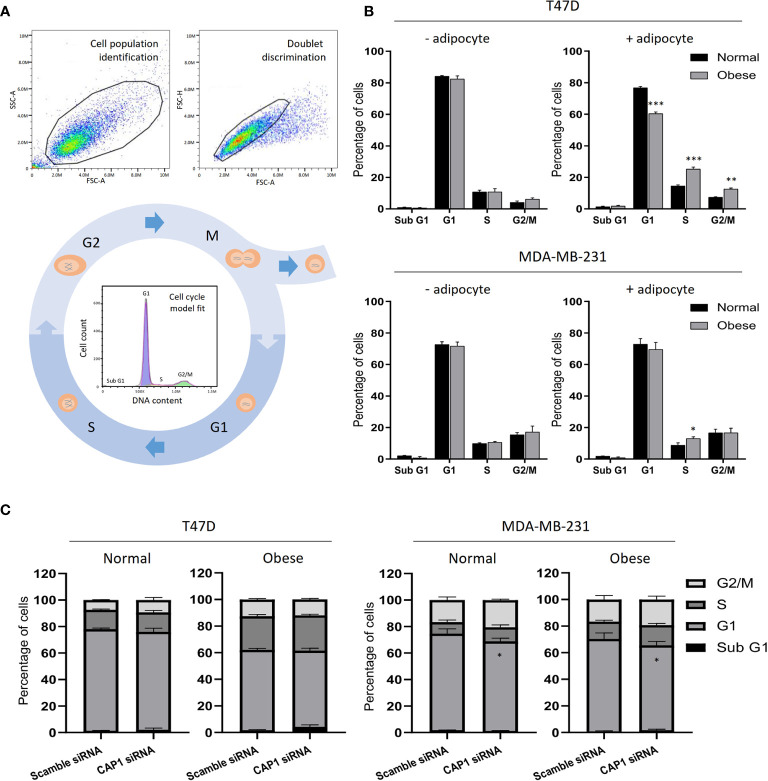
Cell cycle analysis of breast cancer cells. **(A)** Example of flow cytometry gating strategy with cell population identification, doublet discrimination and model fit of the cell cycle phases. **(B)** Cell cycle profiles of T47D and MDA-MB-231 breast cancer cells in the absence or presence of adipocyte secretome under normal and obese conditions. **(C)** Effects by CAP1 knockdown on cell cycle phase distribution in T47D and MDA-MB-231 cells exposed to adipocyte secretome from normal or obese conditions. Graphs show the mean ± SEM from three independent experiments. Multiple comparisons between groups were performed with two-way ANOVA with Sidak’s post-hoc test. *P < 0.05; **P < 0.01; ***P < 0.001.

With the observed modulation of cell proliferation, effects on the cell cycle were subsequently determined by flow cytometry. After gating to obtain a cell population of single cells and automatic DNA histogram analysis (**Figure 5A**), a change to a higher proportion of cells in S- (25.3 vs 14.5%; *P*<0.001) and G2/M-phases (12.6 vs 7.4%; *P*<0.01), and decrease in G1 (60.4 vs 76.7%; *P*<0.001) was observed for T47D cell in response to adipocyte secretome from obese-like, compared with normal conditions (**Figure 5B**). A similar shift was seen in G1 and S-phase for MDA-MB-231 cells (**Figure 5B**). No change in cell cycle phase distribution between normal or obese-like conditions was observed for T47D and MDA-MB-231 cells in the absence of adipocyte stimulation.

Further shifts in the distribution of cells along the phases of the cell cycle were noticed after knockdown of CAP1 (**Figure 5C**). Compared with CAP1 expressing cells, CAP1 silenced T47D exposed to adipocyte secretome from obese-like conditions displayed a relative reduction of cells in G1 (57.2 vs 60.4%), with a concomitant increase in S-phase (26.5 vs 25.3%) and apoptotic sub G1 population (4.3 vs 1.8%), although not reaching statistical significance. Similar findings of lower magnitudes were observed under normal conditions. Compared to CAP1 expressing cells, a lower percentage of cells was observed in G1-phase in CAP1 silenced MDA-MB-231 cells, in response to adipocyte stimulation for both normal (67.6 vs 72.8%; *P*<0.05) and obese-like (63.8 vs 69.5%; *P*<0.05) conditions, whereas a relative accumulation in both S- and G2/M-phase was seen.

### CAP1 Silencing Modified the Protein Phosphorylation Response to Obesity-Associated Adipocyte Secretome Stimulation

**Figure 6:**
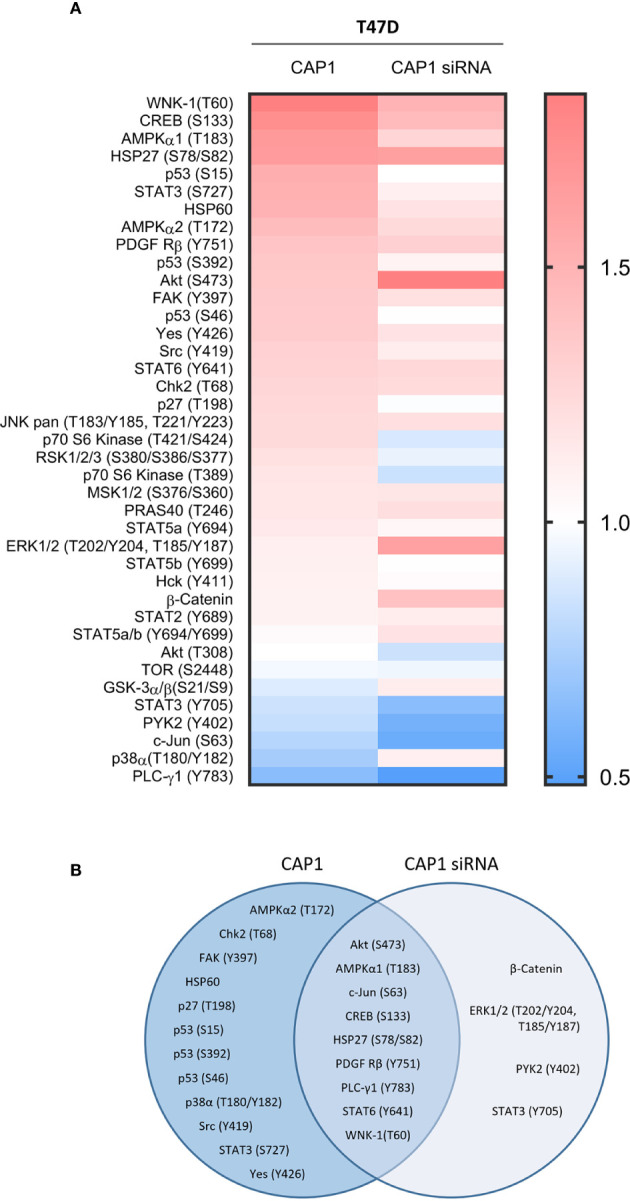
Impact by CAP1 on kinase phosphorylation profiles in T47D breast cancer cells in response to adipocyte secretome from obese versus normal conditions. **(A)** Heatmap displays relative phosphorylation patterns by adipocytes from obesity compared with normal conditions among CAP1 expressing and CAP1 siRNA silenced T47D cells. Relative levels of kinase phosphorylation are shown where levels >1 indicate enhanced phosphorylation and <1 indicate reduced phosphorylation. **(B)** Venn diagram compares the top modulated unique and overlapping protein phosphorylation patterns (up- or downregulated ≥1.25-fold) in response to adipocyte secretome from obese relative to normal conditions between CAP1 expressing or CAP1 silenced T47D cells.

**Figure 7:**
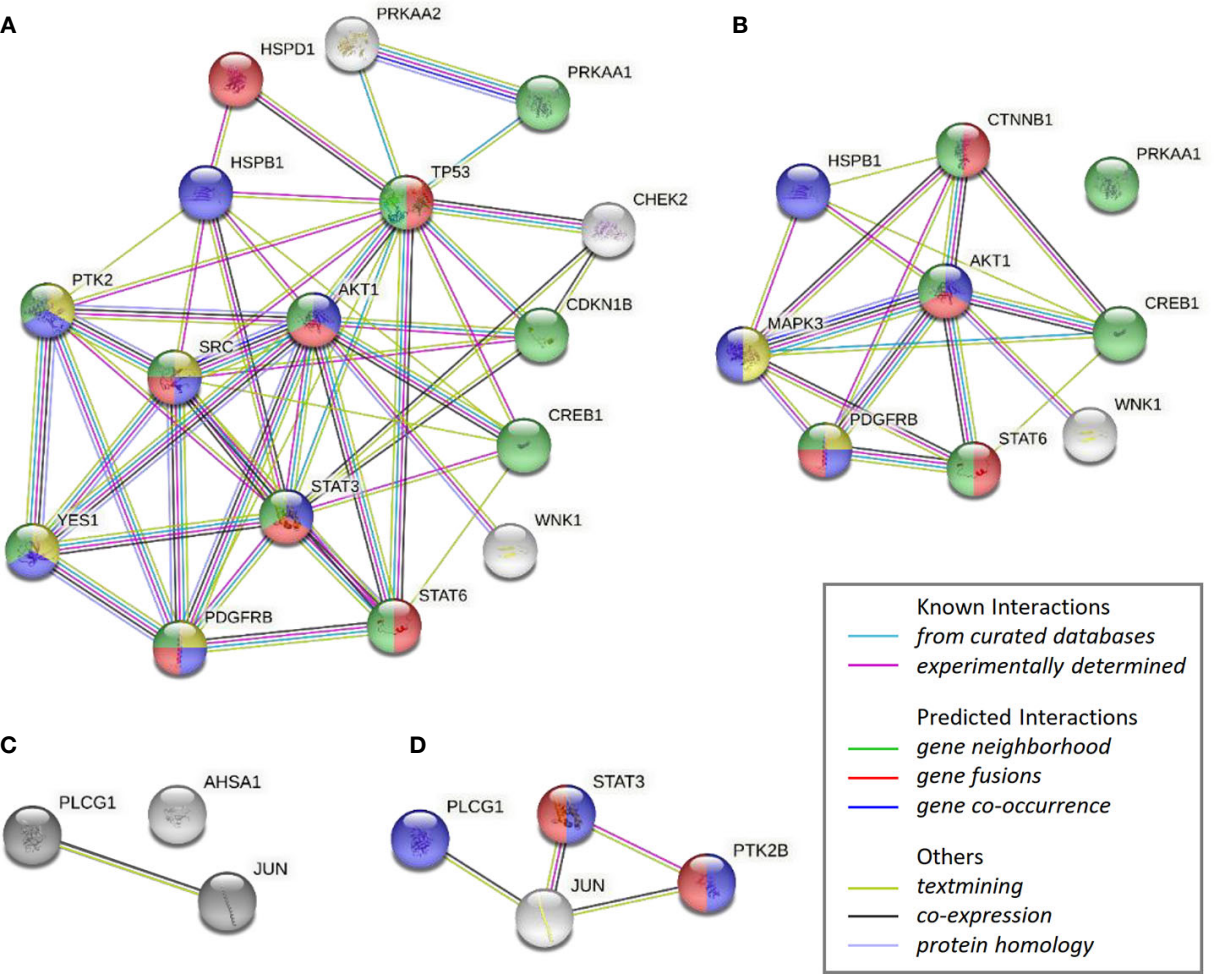
STRING analysis of over-represented biological networks linking phosphorylated kinases modified in T47D cells in response to obese-like relative to normal adipocyte secretome. Interaction networks visualize phosphokinases enhanced in **(A)** CAP1 expressing cells or **(B)** CAP1 silenced cells, and reduced in **(C)** CAP1 expressing cells or **(D)** CAP1 silenced cells. Network nodes show proteins functionally enriched within peptidyl-tyrosine phosphorylation (yellow), transmembrane receptor protein tyrosine kinase signaling (blue), cell population proliferation (red) and regulation of cell population proliferation (green). Edges represent protein-protein associations of known, predicted, or other interactions as indicated.

The impact by CAP1 on protein phosphorylation profiles in response to adipocyte secretome from obese-like relative to normal conditions were assessed among CAP1 expressing and CAP1 silenced T47D cells. Compared to CAP1 expressing cells, the CAP1 silenced cells generally displayed lower expression of assessed phosphorylated proteins (**Figure 6A**). Twelve of the phosphoproteins altered ≥1.25-fold by adipocyte stimulation from obese-like relative to normal conditions, were uniquely changed in CAP1 expressing, but not in CAP1 silenced breast cancer cells (**Figure 6B**). Four proteins had a uniquely altered expression in CAP1 silenced cells, downregulated STAT3 (Y705), and PYK2 (Y402) and upregulated ERK1/2 (T202/Y204, T185/Y187), and β-catenin (**Figure 6B**). Nine phosphoprotein expression patterns were overlapping and modulated in both CAP1 silenced and CAP1 expressing control cells in response to adipocyte secretome from obese-like relative to normal conditions, although with differences in magnitude. To further link changes in pathway activation and potential functional significance of the modified phosphokinases in T47D cells, STRING analysis was applied. The STRING analysis revealed protein-protein interactions and the relationship between modified phosphorylated kinases within identified enriched biological processes in response to obese-like relative to normal adipocyte secretome between CAP1 expressing and CAP1 silenced cells (**Figure 7**). Notably, multiple connections were present among enriched phosphokinases in CAP1 expressing cells (**Figure 7A**) that were weakened following CAP1 silencing (**Figure 7B**).

## Discussion

Obesity is associated with metabolic abnormalities including hyperglycemia and hyperinsulinemia that impact adipocyte functionality with subsequent consequences for breast cancer progression. This study identified phosphorylation patterns of proteins involved in important biological processes enriched in breast cancer cells as a response to the secretome from adipocytes grown under metabolic pressures mimicking obese-like compared to normal conditions. The responsiveness to the obese-like adipocyte secretome translated into enhanced cell proliferation and cell cycle alterations compared with those seen under normal conditions; these specific effects also changed upon knockdown of CAP1. CAP1 silencing further impacted on the adipocyte-induced protein phosphorylation profiles, suggesting a potential role in the molecular mechanisms of obesity-related breast cancer.

It is now widely accepted that excess body fatness is a risk factor for breast cancer, particularly for ER-positive disease in postmenopausal women (36). Several local and systemic effects of obesity have been reported to contribute to these associations including increased estrogen and adipokine biosynthesis, inflammatory mediators, and elevated fasting insulin and glucose levels (3). A recent Mendelian randomization analysis concluded that genetically predicted glucose and insulin-related traits were positively associated with breast cancer risk, results suggesting their role in the obesity-related etiology of breast cancer (37).

In the present study, we demonstrated how the kinase phosphorylation patterns in ER-positive breast cancer cells were affected by the secretome from adipocytes grown under different metabolic conditions. As previously reported, multiple adipokines are modulated and enriched in the adipocyte secretome in response to obese-like compared with normal conditions that can promote cancer cell proliferation and motility (27). The T47D breast cancer cells responded to changes in the environment by increasing the phosphorylation of various kinases when exposed to stimulation by adipocyte secretome derived from obese-like condition. Several of these kinases, including Akt, STATs and Src are activated downstream of growth hormone- and cytokine receptors known to promote cancer proliferation (38), and were enriched within biological processes associated with cell population proliferation.

Herein, WNK-1, the highest upregulated phosphoprotein by the obese-like adipocyte secretome, is activated by Akt, which in turn was found phosphorylated at S473. Both WNK-1 and Akt phosphosignaling are enriched in breast cancer (39). In addition, hyperinsulinemic db/db mice have demonstrated an increased phosphorylation of Akt and WNK1, in line with our results for cells exposed to adipocyte secretome from obese-like conditions (40). The cyclic AMP response element binding protein (CREB), involved in breast cancer progression (41) was activated at S133 in response to obese-like adipocyte secretome stimulation. Interestingly, CREB is driving transcription of aromatase, of particular importance for estrogen production in breast cancer (42). The obese-like adipocyte secretome further stimulated AMPKα phosphorylation, generally considered a nutrient and energy sensor that may hamper energy-requiring processes such as cell division, contrasting to our results (43). In breast cancer, however, peritumoral adipocytes have been shown to release free fatty acids, leading to AMPKα activation through phosphorylation at T183 and metabolic remodeling in breast cancer cells that stimulate their progression and invasiveness (44).

At the cellular level, the adipocyte-derived factors promoted proliferation of both estrogen-receptor positive T47D and estrogen-receptor negative MDA-MB-231 breast cancer cells as previous shown (27). The increase in proliferation was paralleled with a shift towards cell cycle S- and G2/M-phase for cells exposed to adipocyte secretome, particularly from obese-like conditions. WNK-1, has been demonstrated to accelerate G1- to S-phase transition and increase proliferation of rat vascular smooth muscle cells (45). Upon mitogenic stimulation, nuclear p27 is phosphorylated at T197 leading to its degradation or cytoplasmic translocation, events that uncouples its cell cycle inhibitory function and drives cells into S-phase (46), and may link the p27 (T197) phosphorylation and cell cycle progression observed herein.

Silencing of the adipokine receptor and actin-binding protein CAP1, suppressed both basal, and to a greater extent the adipocyte-induced proliferation of both T47D and MDA-MB-231 cells. This was associated with a lower proportion of cells in G1 phase while an accumulation in S-G2/M phases and increase in sub-G1/apoptotic population, particularly under obese-like conditions. This is the first study to demonstrate involvement of CAP1 in adipocyte-mediated breast cancer cell proliferation. CAP1 was recently reported upregulated within a breast cancer risk-associated multi-gene classifying signature enriched in high adipose content breast tissue (47). CAP1 silencing has previously been shown to impact breast cancer cell proliferation and migration in a context dependent manner (32).

Among assessed phosphoproteins altered ≥1.25-fold, 12 were uniquely modulated in CAP1 expressing, but not in CAP1 silenced cells, suggesting their potential involvement in CAP1-associated response to adipocyte stimulation in breast cancer cells. The observed activating phosphorylation of focal adhesion kinase (FAK) at Y397 is known to increase cancer cell viability, growth and motility (48), and several small molecular inhibitors are under development for clinical anticancer therapy. In relation to obesity, the adipokine leptin can induce cell migration and invasion of breast cancer cells in a FAK-Src-STAT3 dependent manner (49). FAK (Y397), STAT3 (Y727), and Src (Y641) were all enhanced in CAP1 expressing cells following obese-like adipocyte stimulation, and may be further involved in CAP1-actin-mediated motility (32). HSP60 expression increases gradually in DCIS to invasive breast cancer tissue and overexpression has been associated with cell proliferation and tumor progression (50). In the present study, an increased expression of HSP60 after obese-like adipocyte secretome stimulation was seen, with relatively lower levels identified in CAP1 silenced cells, which in addition could have contributed to the reduced proliferation in CAP1 knockdown cells. Notably, the obese-like adipocyte secretome additionally enhanced p53 phosphorylation at three residues in CAP1 expressing cells. Tumors frequently display mutated p53 and the T47D cell line carries a p53 missense mutation at L196F that inhibits its ability to form complex with Bxl-2 and induce apoptosis (51), thus the biological consequence of observed phosphorylation herein is unclear.

Four proteins were exclusively modulated ≥1.25-fold by obese-like adipocyte secretome in the absence of CAP1, suggesting cell compensatory mechanisms or CAP1-associated suppressive effects on their regulation. The relative phosphorylation of ERK1/2 after obese-like adipocyte secretome stimulation was higher in CAP1 silenced, compared with CAP1 expressing T47D cells. The relative increase in ERK1/2 phosphorylation, and also Akt (S473), may relate to feedback activation to overcome suppressed growth signals as previously seen with pharmacological mTORC1 inhibitors in breast cancer (52). Similar effects by CAP1 knockdown on ERK1/2 phosphorylation have been demonstrated in the breast cancer cell lines BT-549 and MDA-MB-231 previously, however this resulted in increased proliferation which is in contrast to our results (32). CAP1 silenced cells displayed decreased phosphorylation of PYK2, a member of the FAK family (53). PYK2 is highly expressed in lymph node positive breast cancer and can induce cell proliferation and invasion (53). The decrease of PYK2 (Y402) and the relatively lower phosphorylated levels of its downstream targets Akt, p70S6K, STAT3, FAK and Src in CAP1 silenced cells compared with CAP1 expressing cells after obese-like adipocyte stimulation may in part explain the suppressed proliferation observed.

In conclusion, we designed a culture system to study the effects of adipocyte secretome on CAP1 expressing and CAP1 silenced breast cancer cells. Following exposure of breast cancer cells to adipocyte secretome from conditions mimicking normal physiology and metabolic abnormalities during obesity, we observed a changed cell cycle distribution indicative of cell cycle progression in CAP1 expressing cells and a decreased proliferation for CAP1 silenced cells. We profiled phosphorylation patterns and identified several potential pathways whereby obesity-associated adipocyte secretome and CAP1 may affect cell proliferation and migration in breast cancer. To the best of our knowledge, this is the first study demonstrating that adipocytes under different metabolic conditions change the cell cycle distribution and phosphorylation profile of breast cancer cells and the effect CAP1 has on adipocyte secretome-induced changes. Further mechanistic studies are required to investigate CAP1-associated pathways in obesity-related breast cancer.

## Data Availability Statement

The original contributions presented in the study are included in the article/supplementary material. Further inquiries can be directed to the corresponding authors.

## Author Contributions

Conception and design: MB, AR. Financial support: SB, AR. Data acquisition and analysis: MB. Interpretation of data, manuscript writing or revising it critically for important intellectual content: MB, KE, SB, and AR. All authors contributed to the article and approved the submitted version.

## Funding

This study was supported by the Swedish Cancer Society (CAN 2016/548), the Swedish Research Council (VR 521-2013-2553), Governmental Funding of Clinical Research within the National Health Service (ALF; 2018-Projekt0211), Mrs Berta Kamprad Foundation (FBKS 2018-22 (167), FBKS-2019-48 (219)), the Swedish Breast Cancer Association, Dir Albert Påhlsson Foundation (FB2019-0362), Gyllenstiernska Krapperup Foundation (KR2019-0090), John and Augusta Persson Foundation, the Royal Physiographic Society in Lund, and Lund University APC fund.

## Conflict of Interest

The authors declare that the research was conducted in the absence of any commercial or financial relationships that could be construed as a potential conflict of interest.
